# Effect of Hospital Teaching Status on Mortality and Procedural Complications of Percutaneous Paracentesis in the United States: A Four-Year Analysis of the National Inpatient Sample

**DOI:** 10.7759/cureus.26282

**Published:** 2022-06-24

**Authors:** Mohammad Aldiabat, Yazan Aljabiri, Mohannad H Al-Khateeb, Mubarak H Yusuf, Yassine Kilani, Ali Horoub, Fnu Farukhuddin, Ratib Mahfouz, Adham E Obeidat, Mohammad Darweesh, Mahmoud M Mansour

**Affiliations:** 1 Internal Medicine, NYC (New York City) Health + Hospitals/Lincoln, Bronx, USA; 2 Internal Medicine, Kent Hospital/Brown University, Warwick, USA; 3 Internal Medicine, University of Hawaii, Honolulu, USA; 4 Internal Medicine, East Tennessee State University, Johnson City, USA; 5 Internal Medicine, University of Missouri School of Medicine, Columbia, USA

**Keywords:** outcomes, complications, teaching hospitals, ascites, paracentesis

## Abstract

Objectives

Numerous previous studies investigated the impact of medical training settings on outcomes of hospitalized patients. However, the impact of teaching hospital status on outcomes of percutaneous paracentesis, to the best of our knowledge, has never been studied before.

Methods

Hospitalized patients who underwent percutaneous paracentesis were identified from the National Inpatient Sample database from 2016 to 2019 across the United States (US) teaching and non-teaching hospitals. Outcomes studied were differences in risk of mortality, postprocedural outcomes, and healthcare resource utilization. Multivariate logistic analysis was performed using STATA software (StataCorp LLC, College Station, Texas, US) and results were adjusted for patient and hospital characteristics and comorbidities.

Results

Inpatient mortality rates were significantly higher in patients undergoing paracentesis at US teaching hospitals (adjusted odds ratio (aOR) 1.29, 95%CI 1.23-1.35, p<0.001) compared to non-teaching hospitals. Similarly, higher risk of procedural complications including hemoperitoneum (aOR 1.90, 95%CI 1.65-2.20, p<0.001), hollow viscus perforation (aOR 1.97, 95%CI 1.54-2.51, p<0.001), and vessel injury/laceration (aOR 15.3, 95%CI 2.12-110.2, p=0.007) were noticed in the study group when compared to controls. Furthermore, hospital teaching status was associated with prolonged mean length of stay (9.33 days vs 7.42 days, adjusted mean difference (aMD) 1.81, 95%CI 1.68-1.94, p<0.001) and increased charge of care ($106,014 vs $80,493, aMD $24,926, 95%CI $21,617-$28,235, p <0.001)

Conclusion

Hospitalized patients undergoing paracentesis in US teaching hospitals have an increased risk of mortality, postprocedural complications, prolonged length of stay, and increased charge of care when compared to non-teaching hospitals.

## Introduction

Teaching hospitals provide clinical training required for all prospective physicians, through different residency or fellowship programs. In these settings, patients may be managed by trainee physicians (resident or fellow), under the direct supervision of a faculty member. On the other hand, patients admitted to non-teaching hospitals are usually taken care of by a clinician or provider that is not a trainee. As a result of the theory that training settings carry higher risks of complications and adverse outcomes for patients, secondary to trainees’ inexperience, researchers have long attempted to determine whether medical teaching environments have a significant impact on various patient outcomes than non-teaching settings [1-3]. However, the results are not consistent with a specific pattern of patient benefits [4-6] or harms [3,7].

Percutaneous paracentesis is a commonly performed inpatient procedure as a diagnostic and/or therapeutic intervention for patients with ascites. Given its simplicity, it’s usually performed at the patient’s bedside by residents/fellows in teaching hospital settings. Despite the scarcity of short-term complications of paracentesis (10.5%), especially when the procedure is performed appropriately [8], it includes ascetic fluid leakage (5%), bleeding and vascular laceration (3.3%), hollow viscus perforation (0.4%), and infections (0.2%) [9-11]. In terms of mortality, several studies showed the extreme rarity of deaths among patients undergoing paracentesis (0-0.39%) [8,9,11], with massive bleeding being the main cause of mortality in these cases.

To date, it has not yet been established whether there are differences in outcomes of hospitalized patients undergoing paracentesis based on teaching hospital status. Our study is the first that investigates the differences in outcomes of these patients in terms of mortality, procedural complications including hemoperitoneum, hollow viscus perforation, vessel injury/laceration, in addition to healthcare resources utilization between the United States (US) teaching and non-teaching hospitals. Our proposed hypothesis is that paracentesis outcomes would be negatively impacted in teaching hospitals by the complexity of cases and the inexperience of new trainees who commonly perform this procedure at these centers.

## Materials and methods

Study design and data source

This is a retrospective cohort study utilizing the national inpatient sample (NIS) database from 2016 to 2018, to analyze the outcomes of adult hospitalized patients with ascites who underwent percutaneous paracentesis across US teaching and non-teaching hospitals. The analysis was conducted at the NYC Health + Hospitals/Lincoln, New York, US. This study is adherent to the Strengthening the Reporting of Observational Studies in Epidemiology (STROBE) statement and its checklist [12]. The NIS database is maintained by the Agency for Healthcare Research and Quality's Healthcare Cost and Utilization Project (HCUP), and being the country's largest available all-payer inpatient database, its serves as a representative sample of acute care inpatient hospitalizations across the US [13]. NIS database demonstrated its concordance with other huge databases such as the National Hospital Discharge Survey (NHDS) [14] and has been used as a data source in various clinical epidemiological studies [1,15,16]. The database contains information about each admission, both patient-related and hospital-related.

Participants, eligibility criteria, and exposure

All hospitalized patients in the NIS databases from 2016 to 2019 were identified and screened for adult patients (>= 18 years old) who were admitted and underwent percutaneous paracentesis in both US teaching and non-teaching hospitals and were eligible for inclusion. A teaching hospital is defined per the HCUP as a hospital that has one or more residency programs approved by the Accreditation Council for Graduate Medical Education, is a member of the Council of Teaching Hospitals, or has a ratio of full-time equivalent interns/residents to beds of 0.25 or higher. The International Classification of Diseases, Tenth Revision, Procedure Coding System (ICD-10-PCS) was used to identify percutaneous paracentesis patients using the codes 0W9G30Z, 0W9G3ZX, and 0W9G3ZZ. Patients under the age of 18 years or who were electively admitted to the hospital were excluded from the study, as were those who lacked data for any of the variables in the regression analysis.

Variables

Age, gender, race, expected primary payer, median household income using ZIP code, history of hypertension, diabetes mellitus (DM), smoking, hyperlipidemia, obesity, chronic kidney disease (CKD), coronary artery disease (CAD), peripheral vascular diseases (PVD), chronic obstructive pulmonary disease (COPD), human immunodeficiency virus (HIV), congestive heart failure (CHF), nephrotic syndrome, chronic liver diseases, malignancy, and pancreatitis were among the data collected at the patient level. In addition, the analysis took into account baseline hospital features including hospital region, hospital bed size, and hospital location. Those individuals who were identified with percutaneous paracentesis codes were investigated further to identify those who were coded for procedural complications including hemoperitoneum (K661), hollow viscus perforation (S36), and vessel injury/laceration (S35). A detailed list of the ICD-10 codes utilized to extract the patient's information and comorbidities is given in Appendix 1.

Outcomes

The primary outcome of the analysis was the difference in mortality during the admission at which percutaneous paracentesis was performed between teaching and non-teaching hospitals. Secondary outcomes were procedural complications after paracentesis: hemoperitoneum, hollow viscus perforation, and vessel injury/laceration. We also investigated the difference in length of stay and charge of care in the exposure group when compared to the control, which was estimated based on reported hospital stay cost per HCUP.

Statistical analysis

Stata Statistical Software: Release 17 (2021; StataCorp LLC, College Station, Texas, US), was used to analyze the data. This technique allows researchers to perform studies and report data, variance estimates, and p-values that are objective and nationally representative. In order to get national estimates, weighted samples were used in compliance with HCUP requirements for the use of the NIS database. The chi-square test was used to compare categorical variables (proportions) while the student’s t-test was used to compare continuous variables (mean +/- SD). Unadjusted odds ratios for the main and secondary outcomes were calculated using univariable logistic regression analysis. Afterward, adjusted odds ratios (aOR) for possible confounders were calculated through multivariate logistic regression analysis of age, gender, race, expected primary payer, median household income, past medical history of hypertension, DM, smoking, hyperlipidemia, obesity, CKD, CAD, PVDs, COPD, HIV, hospital region, hospital bed size, and hospital location. Variables associated with significant difference in outcomes on univariate analysis (p-value of less than 0.2) and variables that are considered to be the major drivers of the outcomes of interest regardless of their statistical significance were incorporated into the analysis. In addition, outcomes were adjusted for different etiologies associated with ascites, including CHF, nephrotic syndrome, chronic liver diseases, malignancy, and pancreatitis. With a statistical significance level of 0.05, all p-values were two-sided. The regression analysis was performed on encounters that did not have any missing data/variables.

Ethical considerations

The NIS database only contains retrospective data and does not identify patients. As a result, this study was exempt from Institutional Review Board (IRB) approval.

## Results

Participants characteristics****

**Figure 1:**
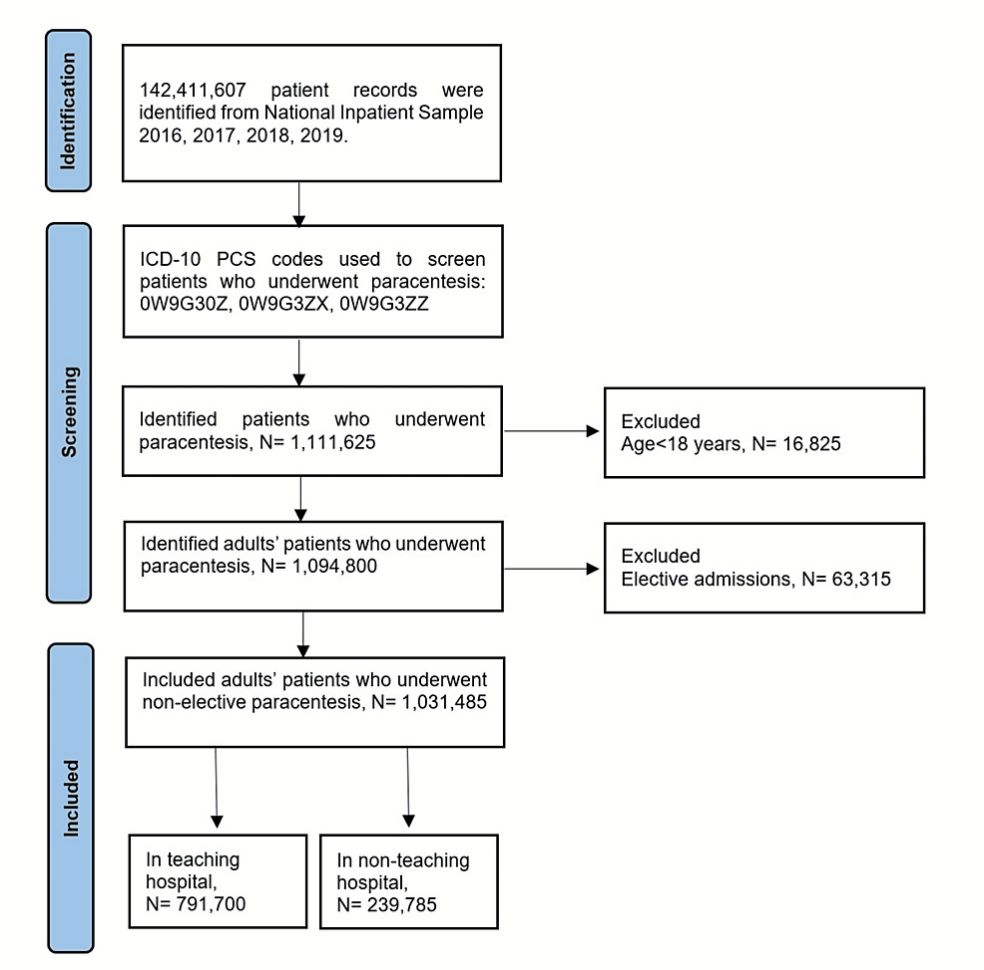
Flow diagram of study sample.

**Figure 2:**
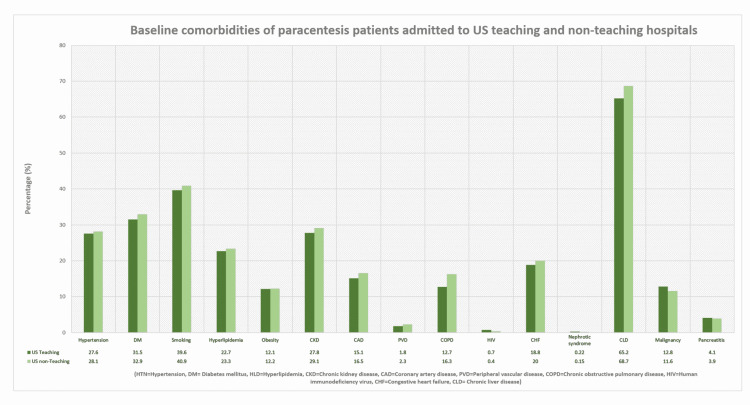
Baseline comorbidities of paracentesis patients admitted to United States (US) teaching and non-teaching hospitals.

Out of a total of 142,411,607 hospitalization records in the NIS 2016-2019 databases, we identified 1,111,625 individuals who underwent percutaneous paracentesis (diagnostic or therapeutic) at one point during their hospitalization’s course. Among these, 1,031,485 were adult patients who underwent the procedure as inpatient, and were included in the study. Subjects <18 years old and those who underwent the procedure on elective basis were excluded. In the study sample, 791,700 (76.8%) subjects were managed at US teaching hospitals, while 239,785 (23.2%) were admitted to non-teaching hospitals as demonstrated in the study flowchart (Figure 1). When compared to non-teaching hospitals, patients who were admitted to teaching hospitals had no clinically significant difference in age (59.0 vs 60.6, p<0.001) or gender distribution (females 43.9% vs 43.3%, p=0.028). However, study group was noticed to have fewer White subjects (64.2% vs 72.1, p<0.001) and more African American (13.8% vs 8.70%, p<0.001). In term of comorbidities (Figure 2), there was no significant difference in prevalence of hypertension (28.1% vs 27.6%, p=0.066), DM (31.5% vs 32.9%, p<0.001), smoking history (39.6% vs 40.9%, p=0.001), hyperlipidemia (22.7% vs 23.3, p=0.039), obesity (12.1% vs 12.2%, p=0.745), CKD (27.8% vs 29.1%, p<0.001), CAD (15.1% vs 16.5%, p<0.001), PVD (1.80% vs 2.30%, p<0.001), COPD (12.7 vs 16.3, p<0.001), and HIV (0.70% vs 0.40%, p<0.001). Similarly, rates of chronic liver diseases (65.2% vs 68.7%, p<0.001), congestive heart failure (18.8% vs 20.0%, p<0.001), nephrotic syndrome (0.22% vs 0.15%, p=0.002), malignancy (12.8% vs 11.6%, p<0.001), and pancreatitis (4.10% vs 3.90%, p=0.028) were not different between both groups of patients as an etiologic factors for development of ascites. Baseline characteristics of patients and hospitals are listed in Table 1.

**Table 1:** Baseline patient and hospital characteristics of subjects undergoing paracentesis.

	Overall %	Teaching %	Non-teaching %	P-value
	N = 1,031,485	N = 791,700 (76.8%)	N = 239,785 (23.2%)	
Patient’s characteristics				
Age, mean years	59.3	59.0	60.6	<0.001
Female	43.8 (451790)	43.9 (347556)	43.3 (103827)	0.028
Racial distribution				
White	66.0 (680780)	64.23 (508509)	72.15 (173005)	<0.001
Black	12.6 (129967)	13.8 (109255)	8.70 (20861)	<0.001
Hispanic	14.4 (148534)	14.7 (116380)	13.3 (31891)	<0.001
Others	2.75 (28366)	2.88 (22801)	2.32 (5563)	<0.001
Insurance type				
Medicaid	46.1 (475515)	45.2 (357848)	49.2 (117974)	<0.001
Medicare	23.6 (243430)	23.9 (189216)	22.8 (54671)	<0.001
Private	24.9 (256840)	25.7 (203467)	22.3 (53472)	<0.001
Uninsured	5.33 (54978)	5.20 (41168)	5.75 (13788)	<0.001
Charlson comorbidity index score				
1	9.47 (97682)	9.09 (71966)	10.7 (25657)	<0.001
2	7.89 (81384)	7.71 (61040)	8.47 (20310)	<0.001
≥3	75.2 (775677)	75.6 (598525)	74.0 (177441)	<0.001
Median annual income, us$				
1–43,999	31.0 (319760)	30.8 (243844)	31.7 (76012)	<0.001
44,000–55,999	26.0 (268186)	25.1 (198717)	28.9 (69298)	<0.001
56,000–73,999	23.9 (246525)	24.1 (190800)	23.0 (55151)	<0.001
≥74,000	19.1 (197014)	20.0 (158340)	16.3 (39085)	<0.001
Hospital characteristics				
Hospital region				
Northeast	18.5 (190825)	20.7 (163882)	11.2 (26856)	<0.001
Midwest	21.7 (223832)	22.8 (180508)	18.2 (43641)	<0.001
South	38.0 (391964)	36.0 (285012)	44.6 (106944)	<0.001
West	21.8 (224864)	20.5 (162299)	26.0 (62344)	<0.001
Hospital bed size				
Small	15.8 (162975)	17.5 (138548)	10.3 (24698)	<0.001
Medium	27.4 (282627)	27.0 (213759)	28.5 (68339)	<0.001
Large	56.8 (585883)	55.5 (439394)	61.2 (146748)	<0.001
Comorbidities				
Hypertension	27.7 (285721)	27.6 (218509)	28.1 (67380)	0.066
Diabetes mellitus	31.8 (328012)	31.5 (249386)	32.9 (78889)	<0.001
Smoking history	39.9 (411563)	39.6 (313513)	40.9 (98072)	0.001
Hyperlipidemia	22.9 (236210)	22.7 (179716)	23.3 (55870)	0.039
Obesity	12.1 (124810)	12.1 (95796)	12.2 (29254)	0.745
Chronic kidney disease	28.1 (289847)	27.8 (220093)	29.1 (69777)	<0.001
Coronary artery disease	15.4 (158849)	15.1 (119547)	16.5 (39565)	<0.001
Peripheral vascular disease	1.87 (19289)	1.80 (14251)	2.30 (5515)	<0.001
Chronic obstructive lung disease	13.5 (139250)	12.7 (100546)	16.3 (39085)	<0.001
Human immunodeficiency virus	0.60 (6189)	0.70 (5542)	0.40 (959)	<0.001
Congestive heart failure	19.0 (195982)	18.8 (148840)	20.0 (47957)	<0.001
Nephrotic syndrome	0.20 (2063)	0.22 (1742)	0.15 (360)	0.002
Chronic liver disease	66.0 (680780)	65.2 (516188)	68.7 (164732)	<0.001
Malignancy	12.5 (128936)	12.8 (101338)	11.6 (27815)	<0.001
Pancreatitis	4.11 (42394)	4.10 (32460)	3.90 (9352)	0.028

Primary outcome: mortality

Among 81,487 (7.90%) estimated deaths in the study population, 65,869 (8.32%) were in the teaching hospitals group and 16,018 (6.68%) were in the non-teaching hospitals group, with a higher aOR for inpatient mortality in patients undergoing paracentesis in teaching hospitals population (aOR 1.29, 95%CI 1.23-1.35, p<0.001) as compared with non-teaching hospitals (Table 2).

**Table 2:** Adjusted odds ratios and percentage of inpatient mortality and procedural complications in paracentesis patients in teaching vs non-teaching hospitals.

	Overall %, n	Teaching %, n	Non-teaching %, n	aOR (95% CI)	P value
Primary outcome					
In‐hospital mortality	7.90 (81487)	8.32 (65869)	6.68 (16018)	1.29 (1.23 - 1.35)	<0.001
Secondary outcomes					
Hemoperitoneum	0.88 (9077)	1.00 (7917)	0.49 (1175)	1.90 (1.65 – 2.20)	<0.001
Hollow viscus perforation	0.36 (3713)	0.04 (317)	0.02 (48)	1.97 (1.54 - 2.51)	<0.001
Vessel injury/laceration	0.03 (309)	0.048 (380)	0.002 (5)	15.3 (2.12 - 110.2)	0.007

Secondary outcomes: procedural complications and healthcare utilization

In terms of secondary outcomes, teaching hospital group was noticed to have significantly higher risk of procedural complications (Table 2) including hemoperitoneum (1.00% vs 0.49%, aOR 1.90, 95%CI 1.65-2.20, p<0.001), hollow viscus perforation (0.04% vs 0.02%, aOR 1.97, 95%CI 1.54-2.51, p<0.001), and vessel injury/laceration (0.048% vs 0.002%, aOR 15.3, 95%CI 2.12-110.2, p=0.007). Additionally, patients who underwent paracentesis in teaching hospitals had prolonged hospital mean length of stay (9.33 days vs 7.42 days, adjusted mean difference (aMD) 1.81, 95%CI 1.68-1.94, p<0.001) and increased hospital charge of care ($106,014 vs 80,493, aMD $24,926, 95%CI $21,617-28,235, p <0.001) when compared to those who underwent the procedure in non-teaching settings (Table 3).

**Table 3:** Adjusted mean difference (aMD) among both study groups in terms of length of stay and hospital charges.

	Overall	Teaching hospital group	Non-teaching hospital group	aMD (95% CI)	P value
Length of stay, mean days	8.89	9.33	7.42	1.81 (1.68 - 1.94)	<0.001
Total hospital charges, mean US$	100,056	106,014	80,493	24,926 (21,617 – 28,235)	<0.001

## Discussion

Key results

Our study's key findings may be summarized as follows: (I) the primary outcome of in-hospital mortality after undergoing percutaneous paracentesis was significantly higher in US teaching hospitals than in non-teaching hospitals; (II) postprocedural complications of paracentesis, including hemoperitoneum, hollow viscus perforation, and vessel injury or laceration were significantly higher in teaching hospitals; (III) Mean length of stay and charge of care for those patients were significantly increased in teaching hospitals compared with non-teaching hospitals. 

Inpatient Mortality in Teaching Hospitals

The most intriguing finding of our study analysis is increased inpatient mortality in individuals who undergo paracentesis in US teaching hospitals compared to those who are managed in non-teaching settings (aOR 1.29, p<0.001). Although prior studies of this association are unavailable, these interesting findings support our proposed theory of the impact of the academic environment on patient outcomes. This difference in mortality can be related to factors including the involvement of trainees in performing the procedure at teaching hospitals, and the complexity of cases encountered at the academic referral centers. However, the proceduralist’s degree of expertise and trainees’ participation seems to have a significant contribution, as the mortality rate remains high in teaching hospitals even after adjusting for various baseline patients’ characteristics, comorbidities, and etiologic factors of ascites, including chronic liver diseases, congestive heart failure, nephrotic syndrome, malignancy, and pancreatitis, among both study groups. Additionally, our findings are supported by the significant correlation between increased risk of procedural complications (see below) and mortality in these patients. Despite the absence of similar studies for comparison, our results are in line with very few previous reports that demonstrate increased in-hospital mortality following procedures performed in academic settings (e.g., endoscopic retrograde cholangiopancreatography) [1] and contradict others that showed no difference (e.g. transcatheter aortic valve replacement) [2] or even improved outcomes (e.g. percutaneous coronary intervention, coronary artery bypass graft) [17,18] in patients undergoing procedures at teaching hospitals.

Procedural Complications

The study has further strengthened our theory, showing a significantly increased risk of procedural complications, including hemoperitoneum (aOR 1.90, p<0.001), vessel injury/laceration (aOR 15.3, p=0.007), and hollow viscus perforation (aOR 1.97, p<0.001) in patients undergoing paracentesis in teaching hospitals. These results are consistent with the increased inpatient mortality in the same study group, reflecting a direct link to the survival rate of those patients. Short-term procedural complications of paracentesis are exceedingly rare [19] and were previously shown to be highly associated with therapeutic than diagnostic paracentesis [9]. The risk of bleeding in advanced liver disease patients with renal failure was shown to be higher before [11]. It is caused by a rupture of an abdominal wall vein or a burst of the mesenteric varices as a result of the abrupt decrease of abdominal wall tension following paracentesis. Given the inability to stratify our study sample based on the severity of liver or kidney diseases, we attribute part of the reported results to the fact that patients managed at teaching hospitals have a more advanced and complicated course of liver and kidney diseases compared to those managed in rural or urban non-teaching hospitals and therefore at higher risk of bleeding. Hollow viscus perforation is rare [9], and can occur when the paracentesis needle pierces the bowel leading to enteric bacteria translocation; however, it’s uncommon to cause overt peritonitis [20].

Length of Stay and Charge of Care

Patients undergoing paracentesis in teaching hospitals were found to have a prolonged length of stay (9.33 days vs 7.42 days) and increased charge of care ($106,014 vs $80,493) when compared to the control group. These values correlate favorably with prior studies that showed increased healthcare resource utilization in US teaching hospitals [21,22]. This can be explained by the need for multiple services (hospital medicine, gastroenterology, cardiology, nephrology, interventional radiology) for the management of complex ascites patients undergoing paracentesis, which are more available for consultation at teaching hospitals than non-teaching leading to increased hospital length of stay and charge of care.

Strengths, limitations, and generalizability

Our study has several strengths. First, it utilizes the largest inpatient database in the US, which ensures the generalizability of the results owing to the huge sample size. It examines a variety of demographics and outcomes of hospitalized patients after adjusting for the most common patient and hospital baseline characteristics to minimize confounding factors. We are aware that our study may have a few limitations. Being the first analysis to study this association, there are no previous studies available for comparison. The NIS is an administrative database that produces retrospective studies, does not provide data on the date of diagnosis or the severity of illness, and uses administrative codes to identify exposures and outcomes, leading to diagnoses being misclassified, under-coded, or over-coded. However, as the misclassification is expected to occur in both study groups equally, it will be considered an error rather than a bias. Errors do not affect the nature of the link between two variables; rather, they make it more difficult to establish statistical significance between them. We cannot claim a causal link between teaching and non-teaching status of hospitals and outcomes of paracentesis since other unmeasured factors may have contributed to the results due to the observational design of the study.

## Conclusions

We studied the link of hospital teaching status with outcomes of ascites patients undergoing paracentesis. Our analysis is significant for increased risk of mortality in patients undergoing paracentesis in US teaching hospitals compared to non-teaching settings. Similarly, procedural complications of paracentesis including hemoperitoneum, vessel injury/laceration, and hollow viscus perforation were higher in hospitals with training programs. Length of stay and cost of care were significantly higher in the study group than in the control group. Being the first study to answer this question, further studies are needed to confirm our findings of postprocedural outcomes in patients undergoing paracentesis in teaching hospitals.

## Appendices

Appendix 1

**Table 4:** All ICD10 codes used in analysis to import patients’ data, exposure, and outcomes ICD10: International Classification of Diseases, Tenth Revision

Percutaneous paracentesis	0W9G30Z, 0W9G3ZX, 0W9G3ZZ
Hypertension	I10
Diabetes mellitus	E11, E10, E09, E08
Smoking	Z87891, F17
Hyperlipidemia	E78
Obesity	E66
Chronic kidney disease	N18
Coronary artery disease	I25
Peripheral vascular disease	I739, I7389
Chronic obstructive pulmonary disease	J40, J41, J42, J43, J44
Human immunodeficiency virus	B20
Congestive heart failure	I50
Nephrotic syndrome	N04
Chronic liver disease	K70, K71, K72, K73, K74, K75, K76, K77
Malignancy	C17, C18, C21, C56, C57, C48, C55, C54, D07, C25, C16, C61, C64, C65, C66, C67, C68, C43, C76, C77, C78, C79, C80, C34, C50
Pancreatitis	K85, K861
Hemoperitoneum	K661
Hollow viscus perforation	S36
Vessel injury/laceration	S35
Acute kidney injury	N17
Sepsis	A41, R65
